# Divergence and Convergence of the Public Health Leadership Competency Framework Against Others in Undergraduate Medical Education: A Scoping Review

**DOI:** 10.3389/phrs.2023.1605806

**Published:** 2023-06-22

**Authors:** Pablo Rodríguez-Feria, Katarzyna Czabanowska, Suzanne Babich, Daniela Rodríguez-Sánchez, Fredy Leonardo Carreño Hernández, Luis Jorge Hernández Flórez

**Affiliations:** ^1^ Institute of Care and Public Health Research, Faculty of Health, Medicine and Life Sciences, Maastricht University, Maastricht, Netherlands; ^2^ Departamento de Salud Pública, Facultad de Medicina, Universidad de los Andes, Bogota, Colombia; ^3^ Department of Health Policy and Management, Institute of Public Health, Faculty of Health Sciences, Jagiellonian University, Krakow, Poland; ^4^ Department of Health Policy and Management, Richard M. Fairbanks School of Public Health, Indianapolis, IN, United States; ^5^ Unidad de Cuidados Intensivos y Críticos del Hospital Universitario Fundación Santa Fe de Bogotá, Bogotá, Colombia; ^6^ Program in Public Health, Schools of Medicine and Government, Universidad de Los Andes, Bogota, Colombia

**Keywords:** undergraduate medical education, leadership, scoping review, competency frameworks, political leadership, leading change, emotional intelligence

## Abstract

**Objective:** The following scoping review is aimed at identifying leadership competency frameworks in Undergraduate Medical Education (UME) by analyzing the thematic scopes, target audiences, and methods involved. A further objective is to compare the frameworks against a standard framework.

**Methods:** The authors extracted the thematic scope and methods of each framework based on the original author’s formulations in each selected paper. The target audience was divided into three sections: UME, medical education, and beyond medical education. The frameworks were converged and diverged against the public health leadership competency framework.

**Results:** Thirty-three frameworks covering thematic scopes such as refugees and migrants were identified. The most common methods to develop leadership frameworks were reviews and interviews. The courses targeted multiple disciplines including medicine and nurses. The identified competency frameworks have not converged among important domains of leadership such as systems thinking, political leadership, leading change, and emotional intelligence.

**Conclusion:** There is a variety of frameworks that support leadership in UME. Nevertheless, they are not consistent in vital domains to face worldwide health challenges. Interdisciplinary and transdisciplinary leadership competency frameworks which address health challenges should be used in UME.

## Introduction

There is an urgent need for health organizations and medical schools to develop competent leaders on every level to effectively address health threats and challenges such as climate change, infectious diseases like COVID-19, and access to medicines, vaccines, and maternal health services, as well as to prepare effectively for emergencies as outbreaks and disrupted health services [1]. In addition, there is a great need for leadership capable of providing preventive and clinical health services to vulnerable populations in conflict and crisis zones [2] by reducing health inequalities, protecting individuals from dangerous products, and creating new innovations in technology, among others [3].

In 2010, Frenk and others stated that teaching leadership to health professionals should be grounded in competency-based curricula that could respond to social needs, adapt to local contexts, and be determined by national stakeholders [4]. Frenk et al advocated for inter-professional (IPE) and trans-professional education (TPE) to break down professional silos and use educational methods that transcend the confines of a classroom (Supplementary Material S1) [4]. In 2020, Thibault went further and proposed six trends for the future of healthcare education, highlighting the importance of preparing professionals for future collaborative practice and shifting to a competency-based time-variable education in order to secure the best prepared practitioners [5]. Competency-based education for medical undergraduate students provides a strong base to develop future doctors by training them alongside other healthcare professionals in leadership [3–5].

Leadership has been recognized as an essential pillar in the education of healthcare professionals, allowing them to excel in healthcare delivery [6], education [3, 6], and public health [7]. Competency-based leadership education grounded on frameworks provides the necessary knowledge, attitudes, values and skills to train physicians, nurses, and public health professionals [3], and supports them in meeting the needs for healthcare in the twenty-first century. Leadership education, especially when properly contextualized, covers a scope of specific health challenges, enhances collaborative work across disciplines, sectors, and organizations [6,7], drives change and effective communication, develops political savvy, and empowers health professionals to develop themselves and others [6,7].

Few studies, however, have critically evaluated leadership competency frameworks, including scopes and contents, used in healthcare education. Webb et al in 2014 [8], James and others in 2021 [9], and Matsas et al in 2022 [10] have reported on teaching leadership in UME using competencies and competency frameworks. Webb concluded that leadership curricula in UME contained a wide range of competencies that were not aligned with any established leadership competency framework [8]. Although they identified a few frameworks used, such as the Canadian Medical Education Directives for Specialists (CanMEDS). The authors recommended aligning leadership curricula with existing leadership frameworks, such as the Medical Leadership Competency Framework (MLCF). Matsas et al found that 52% of the medical schools in the US used a leadership competency framework to create their course content [10]. Webb and Matsas extracted the curricular content and compared it to MLCF [8, 10]. Similarly, James and others used the leadership domains described by Mangrulkar et al [9].

The results of Webb’s and Mangrulkar’s studies do not elaborate on how these leadership frameworks converge and diverge based on a standard framework. Further, Webb, James, and Matsas did not study the thematic scopes of the leadership competency frameworks nor the methodologies used to develop them. They only focused on how leadership is taught in UME, not referring to IPE/TPE. Considering that medical education usually begins at the undergraduate level, it may be important to consider the number of medical schools located around the world. Latin America sits at the top of the list with 513 medical schools, followed by Europe, Africa, and India with 446, 340, and 300, respectively [4].

James et al found a leadership training in Brazil [9], nevertheless this does not provide the methodology to establish leadership skills in UME [11]. Other reviews did not find research in countries with Spanish or Portuguese as official languages [8, 10]. Stakeholders have claimed that future physicians should have leadership competencies in countries such as Brazil, Colombia, Cuba, Chile, Mexico, and Peru [12–19]. Consequently, this review took a closer look at research produced both worldwide and especially in Latin America and the Caribbean (LAC).

This review aims to identify 1) the similarities and differences among leadership competency frameworks used in UME as compared to a “standard” leadership competency framework (the Public Health Leadership Competency Framework Model), 2) their thematic scopes and content, and 3) the methods used to develop them. Furthermore, this study attempts to provide a uniform picture and good practice examples on how to teach leadership in UME to program developers, planners, and health professionals.

## Methods

The research design chosen for this paper was a scoping review. Considering that the body of research on the topic had not been comprehensively reviewed in neither Spanish nor Portuguese, we aimed to identify and analyze knowledge gaps regarding leadership education in UME in LAC. By identifying and mapping available evidence about leadership frameworks and their domains worldwide, we examined methods used to create leadership frameworks in these countries [20].

The Arksey et al, Joanna Briggs Institute’s (JBI), and Levac et al’s guidelines were considered in this review [21–23]. We followed a six-step process including. Step 1) identifying the research question based on the population, concept, and context (PCC) [22]. Step 2) identifying relevant studies by searching index and grey literature in English, Portuguese, and Spanish in six databases between 17 March 2021 and 11 June 2021. The PCC search strategy used controlled vocabulary and the following keywords and their combinations: (population) “students, medical” or “health profe* student*,” (concept) “leadership,” and (context) “curriculum” (Supplementary Material S2).

Step 3) selecting the studies and eliminating duplicates, excluding by title, abstract and full text independently. PRF, FCH, and DRS resolved doubts by a discussion with KC and LJHF. Literature was assessed using methodological quality tools. This review used four tools to appraise quality in quantitative [24], qualitative [25], grey literature [26], and mixed methods studies [27]. Each tool provided three assessment categories: 1) “strong” or “yes,” 2) “moderate,” “I cannot tell” or “?”, and 3) “weak” or “no” (Supplementary Material S3).

Step 4) extracting data based on: title, first author and contact, year of publication, aim, thematic scope, target audience, framework, model, list of competencies or educational objectives (hereby framework), and methods to develop the frameworks. Step 5) checking of the extracted information by two co-authors. All disagreements were reconciled with KC and LJHF. The information was extracted and placed in the chart using verbatim text.

The data on the thematic scope was extracted based on 1) the authors’ purposes and methods used to develop each framework (using the authors´ own words), 2) the stakeholders that were involved in the development of the framework, and 3) the leadership literature that authors considered when creating the frameworks. Authors considered for the methodology section papers were categorized as “strong” or “yes” based on the above-mentioned tools used to measure their quality. Papers with “moderate,” “I cannot tell” or “?”, and papers that were not focused on creating leadership frameworks were not considered. Papers that were categorized as “weak” or “no” in their quality were also excluded from this review. The target audience was categorized in three parts: 1) only UME and/or “medical education” containing UME, 2) residency and fellowship programs, and 3) the category named “beyond medical education” that involves other professions.

Step 6) consulting exercise based on literature quality. The authors of the selected articles were contacted if the results of the quality assessment were rated as “moderate,” “weak,” “I cannot tell,” “no,” or “?”. They were not contacted if the quality was rated as “strong” or “yes.” This hand search and consultation took place from 6 January to 22 April 2022 (Supplementary Material S4).

Finally, the authors chose the Public Health Leadership Competency Framework as the standard based on the following arguments: 1) it has been used to teach leadership in UME in the past, 2) it embodies an interdisciplinary and transdisciplinary approach to leadership education [28–32], 3) it has been successfully used to teach leadership for over a decade [28–32], and 4) it has been validated and used in multiple countries to teach leadership in health-related fields across several education levels [28–32]. Although it was first developed for a public health context, this framework can be considered as universal and it covers areas that are vital for leaders working in the healthcare context, and can therefore be used in UME.

The authors converged and diverged identified frameworks against the standard framework. The first step was to list the frameworks that were mentioned in the studies. Then, the frameworks were divided into two categories: “Leadership was the main focus” or “it was one among other areas of focus.” This was done by analyzing the title, domains, competencies, or objectives. Afterwards, each framework was named F1, F2, to F∞; starting with frameworks in which leadership was the main focus and following those where leadership was one of the areas of the frameworks. Finally, authors extracted the domains, competencies, and objectives of the frameworks to be converged and diverged against the standard competency framework and its domains, based on the definition of each domain: Systems thinking (D1), Political leadership (D2), Collaborative leadership: building and leading interdisciplinary teams (D3), Leadership and communication (D4), Leading change (D5), Emotional intelligence and leadership in team-based organizations (D6), Leadership, organizational learning and development (D7), and Ethics and professionalism (D8).

Convergence meant that the frameworks contained one or more domains from the standard and divergence meant that the frameworks did not overlap with the domains. The results were double checked by a second author. Relative frequency, absolute frequency, central tendency, and variability measurements were calculated per domain. This review was reported via the PRISMA Extension for Scoping Reviews (PRISMA-ScR) [33]; Tableau Public 2021.4 and Microsoft were used to create tables and figures.

## Results

**FIGURE 1:**
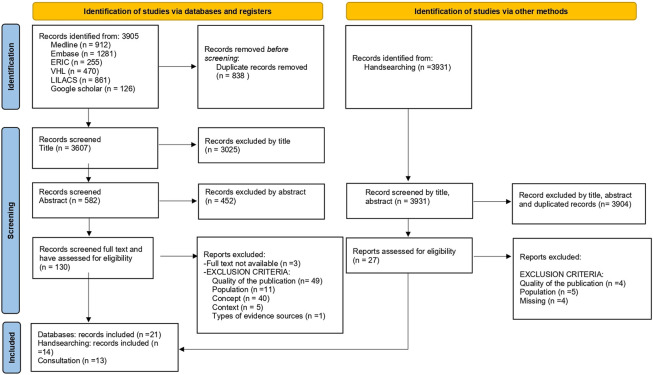
Preferred reporting items for systematic reviews and meta-analyses diagram (worldwide publications. 1970 to 2022).

**FIGURE 2:**
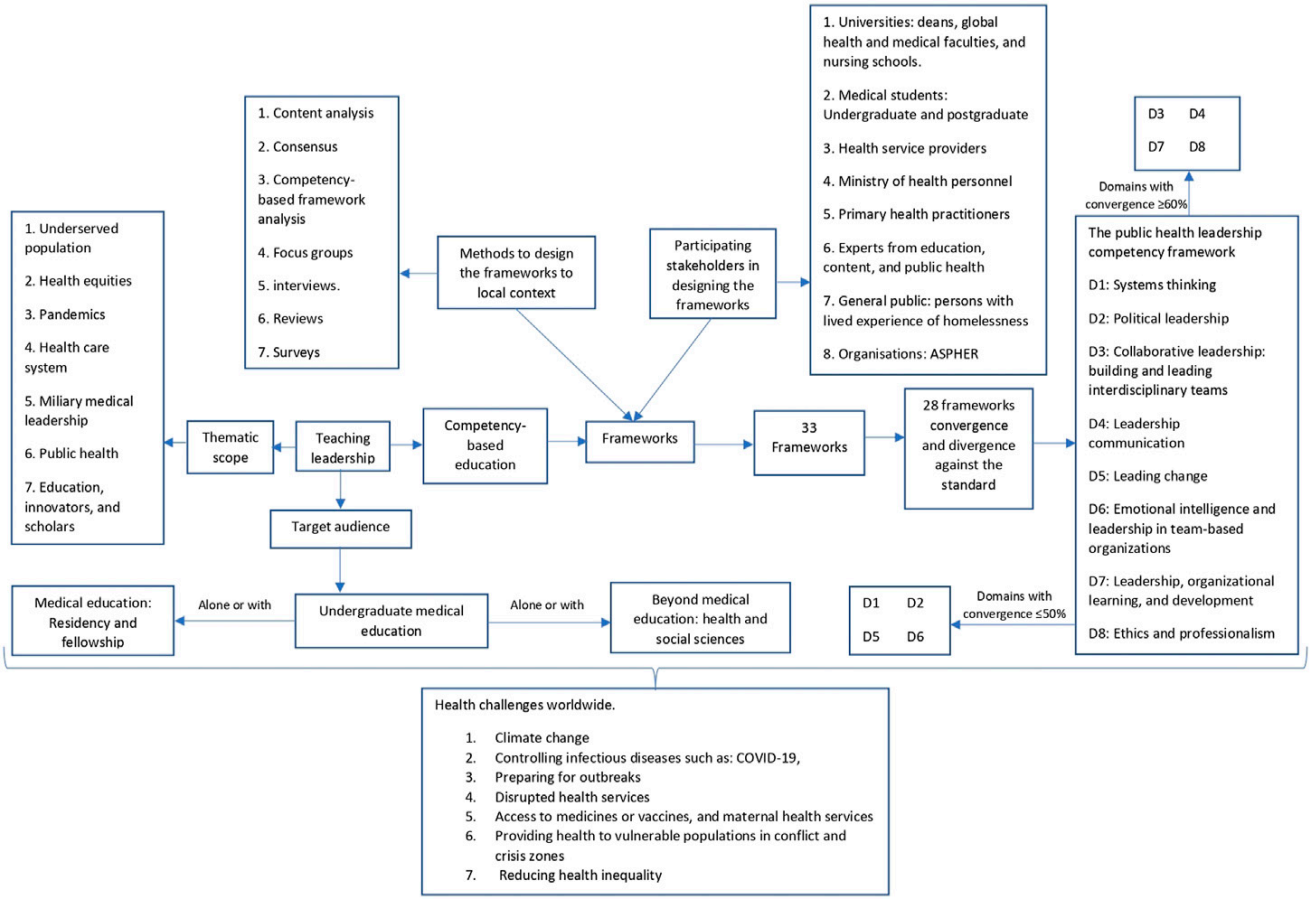
The landscape of leadership frameworks (global perspective. 2009–2022).

**FIGURE 3:**
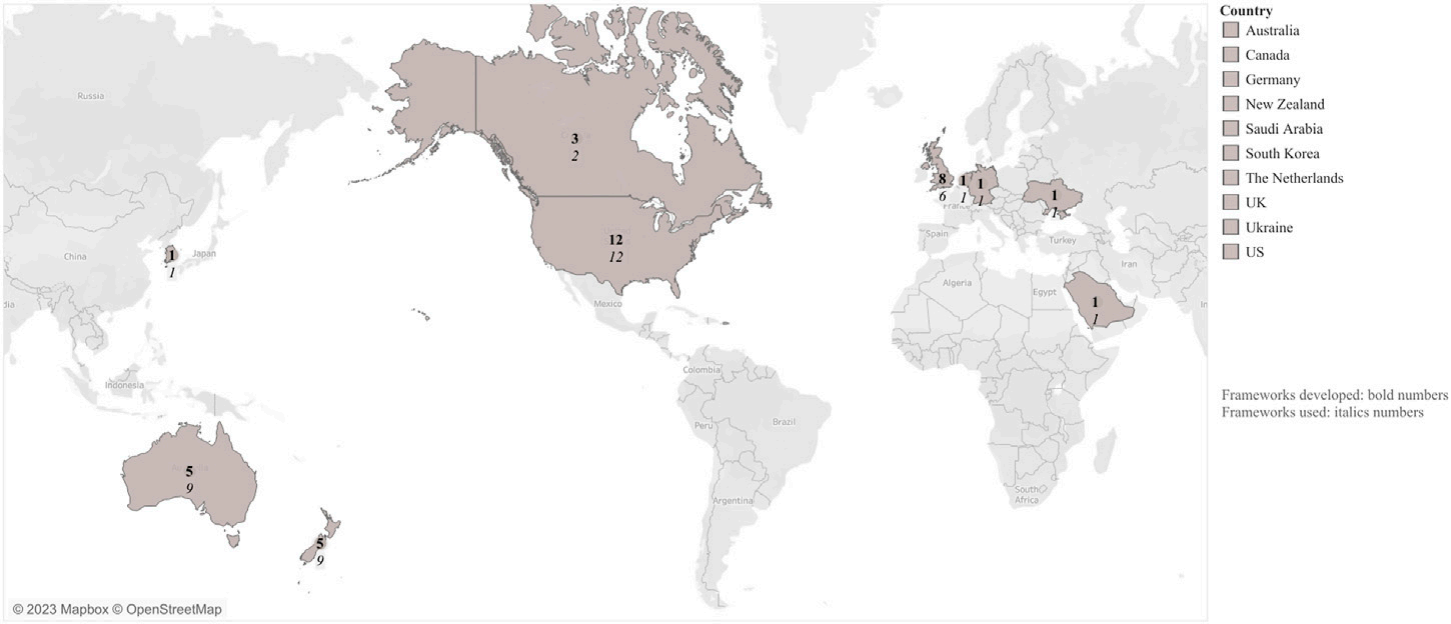
Countries that have developed and used competency-frameworks (Australasian, Europe, Korea, Saudi Arabia, and the Americas. 2009–2022).

Forty-eight documents were considered in this review, representing 23 studies in total [34–56] (Figure 1 and Table 1). The landscape for leadership frameworks in UME including their thematic scopes, target audience, methods to create them, and how they converge and diverge against the standard framework are presented in Figure 2. The 23 studies mentioned 33 frameworks (Supplementary Material S5). The frameworks were created in ten countries, including the United States (US) (12/33, 36%), and the United Kingdom (UK) (8/33, 24%). It is worth noting that countries such as the US, Australia, New Zealand (Australasia), and the UK used more frameworks than others, (*n* = 12), (*n* = 9), and (*n* = 6), respectively (Figure 3). Some studies created their own frameworks (*n* = 19/33, 58%) [34, 36–47, 49, 51, 52, 54–56], while others used frameworks created by someone else [35, 48, 50, 53]. Data extraction for the studies mentioned is available on request.

**TABLE 1:** Summary of reported data items (Australasian, Canada, Europe, Korea, United States, and Saudi Arabia. 2009–2022).

#	Tittle, first author and year	Aim	Thematic scope	Frameworks	Methods to develop frameworks	Target audience
1	An Undergraduate Medical Education Framework for Refugee and Migrant Health: Curriculum Development and Conceptual Approaches [34]	Y	Y	Y	Y	Y
2	Leadership curricula and assessment in Australian and New Zealand medical schools [35]	Y	NA	Y	NA	Y
3	The Pandemic Leadership Model: A Study of Medical Student Values During COVID-19 [36]	Y	Y	Y	Y	Y
4	Contextual Analysis of Stakeholder Opinion on Management and Leadership Competencies for Undergraduate Medical Education: Informing Course Design [37]	Y	Y	Y	Y	Y
5	A student-led curriculum framework for homeless and vulnerably housed populations [38]	Y	Y	Y	Y	Y
6	A first-year leadership programme for medical students [39]	Y	Y	Y	Y	Y
7	Medical Student Consulting: Providing Students Leadership and Business Opportunities While Positively Impacting the Community [40]	Y	Y	Y	Y	Y
8	Medical Student Leader Performance in an Applied Medical Field Practicum [41]	Y	Y	Y	Y	Y
9	Preparing Medical Students to Be Physician Leaders: A Leadership Training Program for Students Designed and Led by Students [42]	Y	Y	Y	Y	Y
10	Identification and evaluation of the core elements of character education for medical students in Korea [43]	Y	Y	Y	Y	Y
11	Leadership and Academic Medicine: Preparing Medical Students and Residents to Be Effective Leaders for the 21st Century [44]	Y	Y	Y	Y	Y
12	On the road to becoming a responsible leader: A simulation-based training approach for final year medical students [45]	Y	Y	Y	Y	Y
13	Health Systems Science Curricula in Undergraduate Medical Education: Identifying and Defining a Potential Curricular Framework [46]	Y	Y	Y	Y	Y
14	The Health Professions Education Pathway: Preparing Students, Residents, and Fellows to Become Future Educators [47]	Y	Y	Y	Y	Y
15	Leadership and management in UK medical school curricula [48]	Y	Y	Y	NA	Y
16	Aspects of development of leader creative thinking of medical student at the undergraduate level of medical education [49]	Y	Y	Y	Y	Y
17	Defining the structure of undergraduate medical leadership and management teaching and assessment in the UK [50]	Y	NA	Y	NA	Y
18	A medical student leadership course led to teamwork, advocacy, and mindfulness [51]	Y	Y	Y	Y	Y
19	Promoting medical students’ reflection on competencies to advance a global health equities curriculum [52]	Y	Y	Y	Y	Y
20	Leadership and management in the undergraduate medical curriculum: a qualitative study of students’ attitudes and opinions at one UK medical school [53]	Y	NA	Y	NA	Y
21	In search for a public health leadership competency framework to support leadership curriculum-a consensus study [54]	Y	Y	Y	Y	Y
22	Preparing students to be academicians: a national student-led summer program in teaching, leadership, scholarship, and academic medical career-building [55]	Y	Y	N	Y	Y
23	Leadership curriculum in undergraduate medical education: a study of student and faculty perspectives [56]	Y	Y	Y	Y	Y

N: Information is not provided, Y: information is provided and NA: information is out of the scope.

### Frameworks: Thematic Scopes and Target Audiences

Universities have used 33 frameworks to teach leadership in UME, nineteen of which contain a thematic scope (Supplementary Material S6) including 1) underserved populations (refugees, migrants and persons who have experienced homelessness), 2) health equities, 3) pandemics, 4) healthcare system, 5) military medical leadership, 6) public health, and 7) education innovators and scholars. Teaching leadership mostly targeted UME (*n* = 26/33), followed by medical education (*n* = 3/33) [43, 44, 47], and beyond medical education (*n* = 3/33) [39, 41, 54] including disciplines such as dentistry, microbiology, and psychology.

### Frameworks: Methods

Thirteen studies included the methodology to contextualize the frameworks in their own settings [34, 36–43, 45, 46, 52, 54, 56] (Table 2 and Supplementary Material S5). Various methods to develop frameworks were used including: 1) reviews (*n* = 8/13, 61%) [34, 38, 39, 41, 42, 46, 52, 54] 2) interviews (*n* = 6/13, 46%) [34, 37, 38, 45, 52, 56], surveys (*n* = 4/13, 30%) [34, 36, 38, 56], and consensus studies (*n* = 3/13, 23%) [38,43,54]. Four studies used three or more methods to triangulate the information [34,38,54,56] in multiple ways. Gruner et al conducted a scoping review alongside interviews, surveys, and a competency-based framework analysis, while Hashmi et al. did a scoping review, surveys, and nominal groups. Furthermore, Czabanowska et al conducted a literature review, a consensus panel, and Delphi survey. Varke et al., on the other hand, used focus groups, interviews, and surveys.

**TABLE 2:** Methodology used to develop leadership frameworks (13/23 studies conducted in Canada, Europe, Korea, United States, and Saudi Arabia. 2009–2022)^a^.

#	Methods First author, year	Content analysis	Consensus	Competency-based framework analysis	Focus group	Interviews	Review	Surveys	Total
1	[34]			X		X	X	X	4
						Semi-structured	A scoping		
2	[36]							X	1
3	[37]					X			1
						Semi-structured			
4	[38]		X				X	X	3
			Nominal group consensus technique				A scoping		
5	[39]					X	X		2
6	[41]						X		1
7	[42]						X		1
8	[43]		X						1
			Delphi survey						
9	[45]					X			1
10	[46]	X					X		2
11	[52]					X	X		2
12	[54]		2X				X		3
			Consensus development panel						
			Delphi survey						
13	[56]				X	X		X	3
						Semi-structured			
Total		1	3	1	1	6	8	4	

^a^ Ten studies did not aim at proving the methods that were used to contextualise a competency-based education to teach leadership in undergraduate medical education. Others their quality assessment was categorised as “moderate,” “I cannot tell,” or “?”. More information can be found in Supplementary Material S5.

These studies report that multiple stakeholders were identified and invited to take part in surveys, interviews, focus groups, and consensus studies. The stakeholders include medical faculties, undergraduate and post-graduate medical students, hospital health service providers, Ministry of Health officials, members of the task force on homelessness, content and education experts, primary healthcare practitioners, public health experts, persons who have experienced homelessness, university deans, education specialists, nursing schools professors, members of the general public, members of the Association of the Schools of Public Health in the European Region (ASPHER), Working Group on good Practice in Public Health Education, marketing, human resources, and health systems administrative leaders.

Studies also report the consultation of diverse literature such as CanMEDS 2015 competency-based framework, The Framework FourCe-PITO (Character, Competence, Context, and Communication across the Personal, Interpersonal, Team and Organizational levels), or the Public Health Leadership Competency Framework to support the development of their frameworks.

### Frameworks: Convergence and Divergence

In some studies, leadership was the main topic of the frameworks (*n* = 15/33, 45%), while it was a subtopic in others (*n* = 14/33, 42%). Some frameworks were not presented in the studies (*n* = 4/33,12%) (Supplementary Material S5). 28 frameworks were analyzed excluding the standard leadership competency framework [44]. Leadership was the main component in half of the frameworks (*n* = 14/28, 50%). Framework # 9 had only one domain that converged with the standard. On the other hand, frameworks #2, #7, and #11 had seven domains in common with the standard. The mean and median were four domains, and the mode three domains (Table 3). The most common convergent domains were D3 (*n* = 10/14, 71%) and D7 (*n* = 10/14, 71%). On the other hand, three domains had ≤50% convergence: D2 (*n* = 3/14, 21%), D1 (*n* = 6/14, 43%), and D5 (*n* = 7/14, 50%).

**TABLE 3:** Convergence^a^ and divergence^b^ of leadership frameworks against the public health leadership competency framework (Australasian, Canada, Europe, Korea, United States, and Saudi Arabia. 2009–2022) [54].

Leadership: Main focus																														
		F1		F2		F3		F4		F5		F6		F7		F8		F9		F10		F11		F12		F13		F14		Total
D1		C		C		D		C		D		C		C		D		D		D		C		D		D		D		6/14
																														43%
D2		C		D		D		D		D		D		D		D		D		D		C		C		D		D		3/14
																														21%
D3		C		C		D		C		C		D		C		C		D		C		C		D		C		C		10/14
																														71%
D4		D		C		C		D		C		C		C		C		D		C		D		D		D		C		8/14
																														57%
D5		C		C		D		D		C		D		C		D		D		D		C		D		C		C		7/14
																														50%
D6		C		C		C		C		D		D		C		C		D		C		C		D		D		C		9/14
																														64%
D7		C		C		C		D		D		D		C		C		C		D		C		C		C		C		10/14
																														71%
D8		D		C		C		D		C		C		C		D		D		D		C		C		D		C		8/14
																														57%
Total		6/8, 75%		7/8, 88%		4/8, 50%		3/8, 38%		4/8, 50%		3/8,38%		7/8, 88%		4/8, 50%		1/8, 13%		3/8, 38%		7/8, 88%		3/8, 38%		3/8, 38%		6/8, 75%		

Leadership is one among other areas of focus																														
	F15		F16		F17		F18		F19		F20		F21		F22		F23		F24		F25		F26		F27		F28		Total	
D1	C		D		C		C		D		C		C		C		C		D		D		D		C		D		8/14	
																													57%	
D2	C		C		D		C		C		C		D		C		C		D		D		C		C		D		9/14	
																													64%	
D3	D		C		C		C		C		C		C		C		C		D		C		C		C		D		11/14	
																													78%	
D4	C		C		C		C		C		C		C		C		D		C		C		D		C		D		11/14	
																													78%	
D5	D		D		C		D		C		C		D		D		C		D		C		D		C		D		6/14	
																													43%	
D6	C		D		C		D		C		C		C		D		D		D		C		D		D		D		6/14	
																													43%	
D7	C		C		C		D		C		C		C		D		C		C		D		D		C		C		10/14	
																													71%	
D8	C		C		C		C		D		C		C		C		C		D		C		C		C		D		11/14	
																													78%	
	6/8, 75%		5/8, 63%		7/8, 88%		5/8, 63%		6/8, 75%		8/8, 100%		6/8, 75%		5/8, 63%		6/8, 75%		2/8, 25%		5/8, 63%		3/8, 38%		7/8, 88%		1/8, 13%			

Domains’ list:

D1: Systems thinking.

D2: Political leadership.

D3: Collaborative leadership: building and leading interdisciplinary teams.

D4: Leadership and communication.

D5: Leading change.

D6: Emotional intelligence and leadership in team-based organizations.

D7: Leadership, organizational learning and development.

D8: Ethics and professionalism.

The frameworks’ list.

F1: Health Leads Australia framework, 2013.

F2: Medical Leadership competency framework Enhancing Engagement in Medical Leadership third Edition, July 2010.

F3: Leadership and management for all doctors, 2012.

F4: Kings Fund: Leadership and Leadership Development in Healthcare (UK), 2015.

F5: The pandemic Leadership Model, 2021.

F6: Portney and Colleagues’ Educational Objectives List.

F7: Leader-Follower Conceptual Framework.

F8: Richard and Colleagues’ list of educational Objectives List.

F9:Dickerman and Colleagues’ List of Educational Objectives.

F10: Schmidt-Humber and Colleagues’ list of competencies.

F11: Healthcare Leadership Model (NHS Leadership Academy, 2013).

F12:Sydorchuk’s list of competencies.

F13: The Leadership Framework (LF) (NHS Leadership Academy 2014).

F14: Varkey Coleman and Colleagues´ list of competencies.

F15: AMC Professionalism and Leadership Graduate Domain and Statement (Australia) 2012.

F16: Good Medical Practice: A Code of Conduct for Doctors in Australia 2014.

F17: CANMEDS 2015.

F18: Refugee Health Curriculum Framework.

F19: Nisreen Rajeh and Colleagues’ competencies list, 2020.

F20: The Homeless Health Education Framework.

F21: The Health Systems Science Framework, 2021–2022.

F22: The 8 Core Categorical Elements of Character Education.

F23:The Health System Science Framework.

F24: Chen et al list of competencies.

F25: Tomorrow’s Doctors (General Medical Council, 2009).

F26:Warde and Colleagues’ List of Competencies.

F27: Mullan and Colleagues’ Framework: Domestic and Global Health Equities.

F28: Coleman and Colleagues’ list of program objectives.

^a^ Converge: C.

^b^ Diverge: D.

Fourteen frameworks contained leadership as a subtopic (*n* = 14/28, 50%). Framework #28 had the lowest convergence with the standard domains, and framework #20 had the highest convergence with eight domains. The mean was five domains, and the median and mode were six domains. The domains that most converged across the frameworks were: D3 (11/14, 78%), D4 (11/14, 78%), and D8 (11/14, 78%). Conversely, two domains had ≤50% in convergence: D5 (*n* = 6/14, 43%) and D6 (*n* = 6/14, 43%).

When comparing the trend of converging domains in the 28 frameworks, political leadership (D2) presented fluctuation from 3 out of 14 frameworks when leadership was the main topic (21%), and it increased to 9 out of 14 frameworks when leadership was a subtopic (64%). D2 was presented in 12/28 frameworks (42%). On the other hand, three domains were constant over the frameworks: leading change (D5) converged ≤50% over the frameworks, collaborative leadership: building and leading interdisciplinary teams (D3), and Leadership, organizational learning, and development (D7) converged ≥71% across the frameworks.

## Discussion

Although during the research process we were not able to identify sources regarding teaching leadership in UME in LAC, Africa, Spain, and Portugal, we were able to find competency frameworks used in medical education ranging from UME to postgraduate studies in different parts of the world. Universities have been using more than 30 leadership frameworks to structure their educational interventions, of which 13 out of 23 mention their methodologies, including one or more methods and diverse stakeholders. Medical leadership has also been offered to other disciplines such as pharmacy and social work. This review describes the most common and uncommon domains of 28 frameworks and compares them to the standard “public health leadership competency framework” [54].

### Major Challenges of Health Professionals Worldwide

The main health challenges previously mentioned must be addressed not only by health professionals, but also by professions related to health such as political and social sciences. In contrast to previous reviews that focused on leadership in medical students [8–10], our study suggests that universities should teach leadership to medical students and their postgraduate programs (residency and fellowship) with an interdisciplinary and transdisciplinary approach. Thus, we believe, future leaders need to acquire competencies related to the collaborative leadership: building and leading interdisciplinary teams (D3) and emotional intelligence and leadership in team-based organizations (D6) domains in order to lay a proper healthcare education environment groundwork for every healthcare team member.

The second challenge in professional education is the rigorous creation and utilization of frameworks for teaching leadership, as it allows for the structuring of competencies by domains and the creation of learning objectives. Furthermore, learning objectives are related to instructional methods, delivery methods, leadership content coverage, and outcome assessment methods. Reviews by Webb et al., Matsas et al., and James et al. address important topics for leadership education such as delivery methods and leadership content. In contrast, our review focuses on the contextualization of leadership frameworks by studying the methodologies used and the stakeholders involved. Additionally, our work showed that leadership frameworks can also be used to teach multiple professions in a multidisciplinary way, allowing them to better face health challenges as global citizens who can see beyond their own countries and local contexts. Systems thinking (D1) will allow leaders to identify and invite actors involved to work together for the sake of our health.

Finally, leadership frameworks allow the comparison of universities’ expertise regarding the teaching of leadership. Nevertheless, the latter possess some difficulties as leadership frameworks are not uniformly used for teaching medical students [8–10]. Subsequently, students need to acquire leadership competencies regarding domains leadership and communication (D4) and leading change (D5) in order to be agents of change in their own organizations. This will allow them to change the *status quo* and promote leadership education.

### Major Challenges of Healthcare Systems Worldwide

Contemporary challenges required health professionals to be equipped with leadership skills to help them effectively address these challenges to improve health and wellbeing of their patients, communities, and societies that they serve. The main global health challenge, identified by the World Health Organization (WHO), is the climate crisis as millions of people die due to air pollution and the transmission of infectious diseases annually [3]. According to two reports by the Economic Commission for Latin America and the Caribbean (ECLAC), 650 million hectares of land have been deforested in the region, which impacts the quality of air, soil, and water. This deforestation also has repercussions on the mortality of humans caused by Malaria, Dengue, and Cholera [57]. Therefore, leaders need a combination of domains such as systems thinking (D1), political leadership (D2) and collaborative leadership: building and leading interdisciplinary teams (D3) that will allow them to identify important health, economic, politic, and social actors needed, in order to create, implement and evaluate initiatives aimed at caring for the environment.

The second challenge identified by the WHO is delivering health in conflict and crisis zones. This organization indicates that global crises and conflicts have led to millions of people leaving their homes and having limited access to healthcare services. Similarly, the third challenge identified by WHO is making healthcare fairer for vulnerable populations based on gender, sexual orientation, and displacement, as they face greater inequity due to their socioeconomic characteristics. In fact, Ruano et al. indicate that being a migrant and refugee is one of the Social Determinants of Health (SDH) that lead to inequity in access and use of healthcare and public health services in LAC [58]. In 2022, the World Migration Report cites 281 million international migrants and 26.4 million refugees [2]. This situation is not unique to LAC, but also occurs in Africa [59, 60], and Europe [61], to name a few examples. The experiences of LAC, African, and European authors show that there are other SDH that affect access and use of healthcare services, such as being a woman, identifying with the LGBTQ+ community, and being part of indigenous communities and ethnic minorities [58]. Future leaders need education in ethics and professionalism in order to advocate for human rights.

### Competency Domains Crucial for Addressing Health Challenges

Introducing leadership education based on leadership competency frameworks can serve as an important vehicle to provide structure for educational interventions. It is of note that the domains which are deemed important for healthcare leaders to tackle crisis situations are not popular in the leadership programs studied. We have found out that the domain (D5) leading change has ≤50% convergence across the 28 studied frameworks. Systems thinking (D1) and political leadership (D2) domains present ≤50% convergence with the frameworks in which leadership is the main focus. Emotional intelligence and leadership in team-based organizations (D6) domain shows ≤50% convergence with the frameworks in which leadership is one among other areas of focus.

It is worth noting that the leading change (D5) domain converged the least with other frameworks although the leadership competencies related to change are crucial in introducing and maintaining innovations and connecting stakeholders who would support them [6,54]. Our study was in line with Mangrulkar et al. who observed that leading change competencies were seldom addressed in medical school curricula, as expressed by the American Medical Association in the Accelerating Change in Medical Education Consortium [62]. Lebano et al. indicate that it is important to consider the available evidence on access to healthcare services for migrants in order to identify access barriers and better plan necessary changes. Leadership is precisely about recognizing challenges and being agents of change, therefore, universities should consider the use of the fifth domain and its competencies. For example, the competency “Serve as a driving force of change, including strategies of change” provides an opportunity for students to be agents of change in the face of current issues.

Systems thinking and political leadership (D1 and D2) are underrepresented across frameworks, and this is unfortunate as leadership is vital for the change and innovation needed to solve the world’s most pressing challenges [63]. These domains and areas are reflected in the current leadership competency needs of health professionals. For example, Czabanowska states that healthcare professionals need political savvy [64,65], and Senge et al claim that they need system thinking to deal with insecurity, and interconnectedness, as well as to be able “to create space for change and enable collective intelligence and wisdom to emerge” [66].

D1 is vital for contemporary healthcare leaders who have to connect, interact with, and proactively foresee the consequences of their decisions on healthcare system performance. In fact, Ruano et al. indicate that actively addressing inequity in access to healthcare in LAC requires going beyond financing the healthcare system and developing health policies that include vulnerable populations so they can access and use healthcare services. Simultaneously, the capacities of healthcare workers need to be strengthened [58].

The Council of Medical Associations of Catalonia (CCMC) believes the promotion of actions aiding the climate emergency to be a public health priority. The Council proposes ten compromises aimed at fighting against the climate emergency. The fourth of these compromises states that “our principal mission is to protect health and we therefore urge local, regional, national and European governments … to begin to legislate, make immediate agreements and take measures that will help slow down climate change and move us away from the emergency situation” [67]. This compromise is closely related to the D2 (Promote the European and national public health agenda) of our framework.

Therefore, universities may consider using competencies that are part of D1 and D2. For example, the first domain has the competency “understand current public health issues and engage in systemic change to address them.” In the future, medical students will understand the economic, social, and political forces in the healthcare system and how they affect vulnerable populations’ access and use of healthcare services. Similarly, the second domain has the competency “advocate and participate in public health policy initiatives at the local, national and/or international levels.” Medical students must participate in the formulation, execution, and evaluation of health policies that guarantee access to and use of healthcare services for these populations.

Another domain with low convergency was emotional intelligence and leadership in team-based organizations (D6). Individuals aspiring to become leaders need to understand their own emotions and how these may affect others [66]. Even more, emotional intelligence (EI) is also critical for work-life balance [68]. In addition, Mintz and Stoller claimed that leadership and EI are desirable and relevant in medical education and practice. Physicians must exhibit EI no matter what their roles are, whether they are executives, academics, or work in healthcare organizations which require them to network with other professions and patients [69].

McCallin et al. studied the relationship between the EI of healthcare workers and the effectiveness of teamwork, which encompasses competencies. The authors concluded that leaders are responsible for team effectiveness and for fostering healthy interactions among team members [70]. Therefore, leaders must understand and manage the emotions of team members, themselves included, and how these relationships impact the team’s effectiveness in healthcare. Universities could contribute to reducing educational silos by including EI in healthcare workers’ training. For example, this domain includes the competency “demonstrate awareness of the impact of your own beliefs, values, and behaviors on your own decision-making and the reactions of others,” which is closely related to what McCallin et al. have referred to.

### Recommendations for Future Study

1. Although healthcare needs related to challenges such as migration, climate change, and conflicts are worldwide, previous reviews [8–10] and this review were not able to identify leadership frameworks that have been rigorously created or contextualized in countries that speak Spanish or Portuguese as an official language in LAC, Africa, and Europe.
2. We invite program developers, planners, and healthcare professionals desiring to teach leadership to document the methods that help identify stakeholders and create leadership competency frameworks.
3. We strongly advise program developers, planners, and healthcare professionals to design, implement, and assess competency frameworks that contain all domains identified in this review.

### Limitations

We covered three out of four official languages in LAC, French was not included. Although we have reviewed and assessed leadership frameworks and have converged and diverged them against a standard that fulfilled our criteria at the time of the study, this review has not searched for informal curricula. To this end, O'Sullivan et al have explored professionalism in medical education by studying the interrelationship between the formal curriculum (on paper), the informal curriculum (in action), and the hidden curriculum (target audience experience) [71].

This review aims to identify 1) the similarities and differences among leadership competency frameworks used in UME as compared to a “standard” leadership competency framework (the Public Health Leadership Competency Framework Model), 2) their thematic scopes and content, and 3) the methods used to develop them. Furthermore, this study attempts to provide a uniform picture and good practice examples on how to teach leadership in UME to program developers, planners, and health professionals.

### Conclusion

There are a variety of frameworks that support leadership in UME. Nevertheless, they are not consistent in vital domains to face worldwide health challenges. Program developers, planners, and healthcare professionals who aim to teach leadership in UME should consider using interdisciplinary and transdisciplinary leadership competency frameworks which include competency domains which are needed to affectively address health challenges of the 21st century and beyond. They can also consider adapting existing frameworks to the specific challenges relevant to their context and system.
