# Supplementary material for: Divergence and Convergence of the Public Health Leadership Competency Framework Against Others in Undergraduate Medical Education: A Scoping Review
Source: Public Health Rev. 2023 Jun 22;44:1605806. doi: 10.3389/phrs.2023.1605806 (PMC10323138; doi:10.3389/phrs.2023.1605806)
Supplement: Supplementary file 1 [file Table1.DOCX]

**Supplementary material 1.** Definitions to be used in this scoping review

| **#** | **Operational concept** | **Definition** | **Reference** |
| --- | --- | --- | --- |
| 3 | Competency. | It is conceptualised as using knowledge, attitudes, sills, and values to benefit the society | Rodríguez-Feria P, Flórez LJH, Czabanowska K. Leadership Competencies for Knowledge Translation in Public Health: A consensus study. J Public Health (Oxf). 2021. <https://doi.org/10.1093/pubmed/fdab286> |
| 4 | competency-based education | Competency-based education (CBE) is an approach to preparing physicians for practice that is fundamentally oriented to graduate outcome abilities and organized around competencies derived from an analysis of societal and patient needs. It deemphasizes time-based training and promises greater accountability, flexibility, and learner-centredness. | Frank JR, Mungroo R, Ahmad Y, Wang M, De Rossi S, Horsley T. Toward a definition of competency-based education in medicine: a systematic review of published definitions. Med Teach. 2010;32(8):631-7. |
| 5 | Framework | Frameworks may be described as analytic (e.g. knowledge, skills, attitudes), synthetic (e.g. focused on clinical activities) and developmental (e.g. beginner, competent, expert) and often have a hybrid nature. | Pangaro L, ten Cate O. Frameworks for learner assessment in medicine: AMEE Guide No. 78. Medical Teacher. 2013; 35(6): e1197-e1210. [frameworks-for-learner-assessment-in-medicine.pdf (marshall.edu)](https://jcesom.marshall.edu/media/53467/frameworks-for-learner-assessment-in-medicine.pdf) |
| 6 | Interprofessional education. | involves students of two or more professions learning together, especially about each other’s roles | Frenk J, Chen L, Bhutta ZA, Cohen J, Crisp N, Evans T, et al. Health professionals for a new century: transforming education to strengthen health systems in an interdependent world. Lancet. 2010;376(9756):1923-58. |
| 7 | Medical Education | The process of teaching, learning and training of students with an on-going integration of knowledge, experience, skills, qualities, responsibility and values which qualify an individual to practice medicine. It is divided into undergraduate, postgraduate and continuing medical education, but increasingly there is a focus on the "lifelong" nature of medical education | The Euroasian Centre for Accreditation and Quality Assurance in Higher Education and Health Care. Available from: [Glossary of Medical Education Terms (engl).pdf (ecaqa.org)](http://www.ecaqa.org/doxs/Glossary%20of%20Medical%20Education%20Terms%20(engl).pdf) Accessed on April 4^th^ 2022. |
| 8 | Postgraduate Medical Education | Postgraduate education, graduate medical education or specialty training is used to designate the more or less continuous period of post-basic training which, when it occurs, normally directly follows undergraduate training and is designed to lead to competence in a chosen branch of medical practice | The Euroasian Centre for Accreditation and Quality Assurance in Higher Education and Health Care. Available from: [Glossary of Medical Education Terms (engl).pdf (ecaqa.org)](http://www.ecaqa.org/doxs/Glossary%20of%20Medical%20Education%20Terms%20(engl).pdf) Accessed on January 24^th^ 2023. |
| 9 | Transprofessional education | That includes non-professional health workers might be of even greater importance for health-system performance, especially the teamwork of professionals with basic and ancillary health workers, administrators and managers, policy makers, and leaders of the local community. | Frenk J, Chen L, Bhutta ZA, Cohen J, Crisp N, Evans T, et al. Health professionals for a new century: transforming education to strengthen health systems in an interdependent world. Lancet. 2010;376(9756):1923-58. |
| 10 | Undergraduate Medical education. | the period beginning when a student enters medical school and ends with the final examination for basic medical qualification. This period of education comprises a pre-clinical and a clinical period. | The Euroasian Centre for Accreditation and Quality Assurance in Higher Education and Health Care. Available from: [Glossary of Medical Education Terms (engl).pdf (ecaqa.org)](http://www.ecaqa.org/doxs/Glossary%20of%20Medical%20Education%20Terms%20(engl).pdf) Accessed on January 24^th^ 2023. |
