# Supplementary material for: Divergence and Convergence of the Public Health Leadership Competency Framework Against Others in Undergraduate Medical Education: A Scoping Review
Source: Public Health Rev. 2023 Jun 22;44:1605806. doi: 10.3389/phrs.2023.1605806 (PMC10323138; doi:10.3389/phrs.2023.1605806)
Supplement: Supplementary file 2 [file Table2.DOCX]

**Supplementary material 2. Scoping review: search strategy.**

**Step 1: Search strategy in two databases**

Webb et al’s systematic review search strategy was used to do the first step. PubMed’s strategy was: "Students, Medical [MeSH] AND leadership [MeSH Terms] or leadership [all field] AND curriculum". This strategy did not mention if curriculum was a "MeSH", "Title and abstract" or "all field".

Medline via PubMed.

| **Date of search** | **March 17th 2021** |
| --- | --- |
| Search String | ((LEADERSHIP) AND (Students, Medical[MeSH Terms])) AND (Curriculum[MeSH Terms]) |
| Analysis of the text words and index terms contained in the title and abstract.  Mesh words were selected based on the [National Center for Biotechnology Information ( NCBI).](https://www.ncbi.nlm.nih.gov/) | Index terms:  -Population: Students, Medical [MeSH terms].  -Concept: Leadership [ All field] and Curriculum [MeSH terms].  -Context: Not applicable.  Tittle and abstract: The search strategy did not contain tittle and abstract domains. |
| Hits | 168 results |

**LILACS ( Search Strategy in Spanish)**

| **Date of search** | **March 17th 2021** |
| --- | --- |
| Search String | Estudiantes [Categoria DeCS] and liderazgo [Palabras] and curriculum [Categoria DeCS] |
| Analysis of the text words and index terms contained in the title and abstract.  Titles and abstracts can be in English, Spanish and/or Portuguese. | Search Strategy 1  -Population: Estudiantes (DeCS)  -Concept: Liderazgo (palabras) and Curriculum (DeCS)  -Context: Not applicable. |
| Hits | 0 results |

**Step 2: Search strategy in each database.**

This review selected six databases

1. Medline via PubMed,
2. Embase via Ovid,
3. Education Resources Information Center,
4. Virtual Health Library,
5. Health Information from Latin America and the Caribbean Countries, and
6. Google scholar.
7. **Medline via PubMed.**

| **Date of search** | **June 4th 2021.** |
| --- | --- |
| Search String | 1.Students, Medical [Mesh].  2.Medical student* [ ti/ab].  3.Health profe* student* [ti,ab].  4.Public health profe* [ti,ab].  5.#1 OR # 2 OR # 3 OR # 4 OR #5  6.Leadership [ Mesh].  7.Leadership [ti/ab].  8.#6 OR # 7  9.Curriculum [ Mesh]  10.Competency-Based Education [ Mesh]  11.Education, Public Health Professional [Mesh]  12.Education, Medical [MeSH]  13.competency framework [ti/ab]  14.competency-based education [ ti/ab].  15.undergraduate medical education [ti/ab]  16.Education, Medical, Undergraduate [MeSH]  17. #9 OR # 10 OR # 11 OR # 12 OR #13 OR #14 OR #15 OR #16  18.Language Spanish  19.Language English  20.Language Portuguese  21.#18 OR #19 OR #20  22.# 5 AND #8 AND # 17 AND # 21 AND Date publication 1/1/1970 to present  ((((((((Students, Medical[MeSH Terms])) OR (Medical student*[Title/Abstract])) OR (Health profe* student*[Title/Abstract])) OR (Public health profe*[Title/Abstract])) AND ((Leadership[MeSH Terms]) OR (Leadership[Title/Abstract]))) AND ((((((((Curriculum[MeSH Terms]) OR (Competency-Based Education[MeSH Terms])) OR (Education, Public Health Professional[MeSH Terms])) OR (Education, Medical[MeSH Terms])) OR (competency framework[Title/Abstract])) OR (competency-based education[Title/Abstract])) OR (undergraduate medical education[Title/Abstract])) OR (Education, Medical, Undergraduate[MeSH Terms]))) AND (((Spanish[Language]) OR (English[Language])) OR (Portuguese[Language]))) AND (("1970/01/01"[Date - Publication] : "3000"[Date - Publication])) |
| Analysis of the text words and index terms contained in the title and abstract.  Mesh words were selected based on the [National Center for Biotechnology Information ( NCBI).](https://www.ncbi.nlm.nih.gov/) | Index terms:  -Population: Students, Medical [Mesh].  -Concept: Leadership [ Mesh].  -Context: Curriculum [ Mesh],Competency-Based Education [ Mesh] Education, Public Health Professional [Mesh], Education, Medical [MeSH] and Education, Medical, Undergraduate.  Tittle and abstract:  -Population: Medical student* ( ti/ab), health profe* students ( ti,ab), Public health profe* ( ti,ab).  -Concept: leadership ( ti/ab).  -Context: competency framework ( ti/ab), competency-based education ( ti/ab), undergraduate medical education [ti/ab] |
| Hits | 912 |

1. Embase via Ovid ( 1974-2021)

| **Date of search** | **June 4th 2021.** |
| --- | --- |
| Search String | 1.medical student [Subject Heading]  2.health student [subject heading]  3.Medical student* ti  4.Medical student* ab  5.health profe* student* ti.  6.health profe* student* ab.  7.public health profe* ti  8.public health profe* ab  9.#1 OR # 2 OR # 3 OR # 4 OR #5 OR # 6 OR #7 OR # 8  10.Leadership [Subject Heading].  11.leadership ti.  12.leadership ab.  13.#10 OR # 11 OR # 12  14.medical education [ Subject Heading]  15.curriculum[ Subject Heading]  16.competency-based education ti  17.competency-based education.ab.  18.curriculum ti.  19.curriculum ab.  20.undergraduate medical education ti  21.undergraduate medical education ab  22.#14 OR # 15 OR # 16 OR # 17 OR #18 OR # 19 OR #20 OR # 21  23.Spanish.lg  24.English.lg  25.Portuguese .lg  26.# 23 OR # 24 OR # 25  27.#9 AND # 13 AND # 22 AND # 26  (medical student or health student).sh. or Medical student*.ti. or Medical student*.ab. or health profe* student*.ti. or health profe* student*.ab. or public health profe*.ti. or public health profe*.ab.  leadership.sh. or leadership.ti. or leadership.ab.  (medical education or curriculum).sh. or competency-based education.ti. or competency-based education.ab. or curriculum.ti. or curriculum.ab. or undergraduate medical education.ti. or undergraduate medical education.ab.  (spanish or english or portuguese).lg.  a and b and c and d |
| Analysis of the text words and index terms contained in the title and abstract.   Emtree (Embase subject headings) | Index terms:  -Population: medical student [Subject Heading], health student [subject heading]  -Concept: Leadership [Subject Heading].  -Context: medical education [ Subject Heading], curriculum[ Subject Heading],  Abstract:  -Population: Medical student*ti.ab., health profe* student* ti.ab., public health profe* ti.ab.  -Concept: leadership ti.ab.  -Context: competency-based education ti.ab., curriculum ti.ab., undergraduate medical education ti.ab. |
| Hits | 1281 |

1. **ERIC via EBSCOhost**

| **Date of search** | **6th June 2021** |
| --- | --- |
| Search String | 1.KW medical students  2.TI public health student  3.AB public health student  4.TI health student  5.AB health student  6. #1 OR #2 OR #3 OR #4 OR #5  7.TX leadership  8.TX education  9.TX training leadership  10. #8 OR #9  11. #6 AND #7 AND #10  ( KW medical students OR TI public health student OR AB public health student OR TI health student OR AB health student ) AND TX leadership AND TX ( education or traning leadership )  Limiters - Date Published: 19700101-20211231 |
| Analysis of the text words and index terms contained in the title and abstract. | terms:  -Population: KW medical students, TI public health student, AB public health student, TI health student , AB health student  -Concept: TX leadership  -Context: TX education, TX training leadership |
| Hits | 255 |

1. **Virtual Health Library**

| **Date of search** | **6th June 2021.** |
| --- | --- |
| Search String | Titulo, resumen y asunto: (estudiante) AND (liderazgo) AND (educación ) AND ( la:("en" OR "pt" OR "es")) AND (year_cluster:[1970 TO 2021]) |
| Analysis of the text words and index terms contained in the title and abstract. | Search Strategy 1  -Population: estudiante  -Concept: liderazgo  -Context: Educación |
| Hits | 470 |

1. **LILACS ( Search Strategy in Spanish)**

| **Date of search** | **6th June 2021** |
| --- | --- |
| Search String | Estudiante[ palabra] AND liderazgo [ palabra] AND educación [ palabra] |
| Analysis of the words | Search Strategy 1  -Population: estudiantes (palabras)  -Concept: Liderazgo (palabras).  -Context: Educación(palabras) |
| Hits 1 ( too narrow the search strategy) ( excluded) | 74 |
| Date of Search | June 9th 2021 |
| Search String | Liderazgo [palabra] and educación or educación basada en competencias. [palabra] |
| Analysis of the words | Search Strategy 1  -Population: No included  -Concept: Liderazgo (palabras).  -Context: Educación(palabras) or educación basada en competencias (palabras). |
| Hits 2 final search ( included ) | 440 |

1. **LILACS ( Search Strategy in Portuguese )**

| **Date of search** | **6th June 2021.** |
| --- | --- |
| Search String | estudantes [ palabra] AND Liderança [ palabra] AND Educaçao [ palabra] |
| Analysis of the text words. | Search Strategy 1  -Population: estudantes (palavras)  -Concept: Liderança (palavras).  -Context: Educaçao (palavras) |
| Hits 1 too narrow the search strategy ( excluded) | 66 |
| Date of search | 9th June 2021 |
|  | -Population: No aplicable  -Concept: Liderança (palavras).  -Context: Educaçao (palavras) or Educaçao baseada em competencias |
| Hits 2: final search ( included) | 421 |

**7. Google Scholar**

| **Date of search** | **11th June 2021** |
| --- | --- |
| Search String 1 | Find articles  with all of the words: health students  with the exact phrase: leadership  with at least one of the words: education, curriculum, training  without the words: Not applicable  where my words occur:  Return articles dated between: 1970-2021  Search English, Portuguese, Spanish |
| Analysis of the text words. | -Population: health students  -Concept: leadership  -Context: education, curriculum, training |
| Hits 1 ( included) | 11 |
| Search String 2 | Find articles  with all of the words: health professions  with the exact phrase: leadership  with at least one of the words: education, curriculum, training  without the words: Not applicable  where my words occur:  Return articles dated between: 1970-2021  Search English, Portuguese, Spanish |
| Analysis of the text words | -Population: health students  -Concept: leadership  -Context: education, curriculum, training |
| Hits 2 ( included) | 20 |
| Search String 3 | Find articles  with all of the words: public health  with the exact phrase: leadership  with at least one of the words: education, curriculum, training  without the words: Not applicable  where my words occur:  Return articles dated between: 1970-2021  Search English, Portuguese, Spanish. |
| Analysis of the text words | -Population: public health  -Concept: leadership  -Context: education, curriculum, training. |
| Hits 3 ( included) | 95 |

Total hits and last search 11th June 2021.

| 912 + 1281 + 255 + 470+ 440 + 421 + 11+ +20+ 95 = 3905 |
| --- |
