# Supplementary material for: Divergence and Convergence of the Public Health Leadership Competency Framework Against Others in Undergraduate Medical Education: A Scoping Review
Source: Public Health Rev. 2023 Jun 22;44:1605806. doi: 10.3389/phrs.2023.1605806 (PMC10323138; doi:10.3389/phrs.2023.1605806)
Supplement: Supplementary file 3 [file Table5.docx]

Supplementary file 5: Frameworks mentioned in this review based on including leadership focus, quality assurance, and reference (global literature: 2009-2022)

| **#** | **Frameworks** | **Is leadership the main focus?** | **Quality assurance when developing a framework** | **Reference** |
| --- | --- | --- | --- | --- |
| 1 | The Refugee Health Curriculum Framework,2021^^[[1]](#footnote-1)^^ | N | Quality assessment: Strong or yes | Gruner  2022 |
| 2 | AMC Professionalism and Leadership Graduate Domain and Statement (Australia) 2012 | N | This study was an overview, and assessing the framework´s methodology was out of scope | Ross  2021 |
| 3 | Good Medical Practice: A Code of Conduct for Doctors in Australia 2014 | N | This study was an overview, and assessing the framework´s methodology was out of scope | Ross  2021 |
| 4 | Health Leads Australia Framework 2013 | Y | This study was an overview, and assessing the framework´s methodology was out of scope | Ross  2021 |
| 5 | Medical Leadership Competency Framework, 2010 | Y | This study was an overview, and assessing the framework´s methodology was out of scope | Ross  2021 |
|  |  | Y | This study was an overview, and assessing the framework´s methodology was out of scope | Jefferies  2016 |
| 6 | CanMEDS: Better Standards, Better Physicians, Better Care (Canada) 2015 | N | This study was an overview, and assessing the framework´s methodology was out of scope | Ross  2021 |
| 7 | Australian Commission on Safety and Quality in Health Care^^[[2]](#footnote-2)^^ | Missing | This study was an overview, and assessing the framework´s methodology was out of scope | Ross  2021 |
| 8 | Leading and Managing Health Services: An Australian Perspective 2015^^[[3]](#footnote-3)^^ | Missing | This study was an overview, and assessing the framework´s methodology was out of scope | Ross  2021 |
| 9 | General Medical Council: Leadership and Management for Doctors (UK) 2012 | Y | This study was an overview, and assessing the framework´s methodology was out of scope | Ross  2021 |
| 10 | Kings Fund: Leadership and Leadership Development in Health Care (UK) 2015 | Y | This study was an overview, and assessing the framework´s methodology was out of scope | Ross  2021 |
| 11 | The Pandemic Leadership Model, 2021. | Y | Quality assessment: Strong or yes | Bernard  2021 |
| 12 | [Nisreen Rajeh](https://pubmed.ncbi.nlm.nih.gov/?term=Rajeh+N&cauthor_id=32844118) and colleagues’ competencies list, 2020. | N | Quality assessment: Strong or yes | Rajeh  2020 |
| 13 | The Homeless Health Education Framework^^[[4]](#footnote-4)^^. | N | Quality assessment: Strong or yes | Hashmi  2020 |
| 14 | The Health Systems Science Framework, 2021-2022. | N | Quality assessment: Strong or yes | Wagenschutz  2019 |
| 15 | Portney and colleagues´ educational objectives list. | Y | Quality assessment: moderate, I cannot tell or ? | Portney  2019 |
| 16 | Leader-Follower conceptual framework | Y | Quality assessment: Strong or yes | Barry  2019 |
| 17 | Richard and colleagues’ list of educational objectives | Y | Quality assessment: Strong or yes | Richard  2019 |
| 18 | The 8 core categorical elements of character education. | N | Quality assessment: Strong or yes | Hur  2019 |
| 19 | Dickerman and colleagues’ list of educational objectives. | Y | Quality assessment: moderate, I cannot tell or ? | Dickerman  2018 |
| 20 | Schmidt-Huber and colleagues’ list of competencies. | Y | Quality assessment: Strong or yes | Schmidt-Huber  2017 |
| 21 | The health systems science (HSS) curricular framework. | N | Quality assessment: Strong or yes | Gonzalo  2017 |
| 22 | Chen et al list of competencies. | N | Quality assessment: moderate, I cannot tell or ? | Chen  2017 |
| 23 | Healthcare Leadership Model (NHS Leadership Academy, 2013)- | Y | This study was an overview, and assessing the framework´s methodology was out of scope | Jefferies  2016 |
| 24 | Tomorrow’s Doctors (General Medical Council, 2009) | N | This study was an overview, and assessing the framework´s methodology was out of scope | Jefferies  2016 |
| 25 | the Faculty of Medical Leadership and Management’s (FMLM) Standards for Medical Leadership (Faculty of Medical Leadership and Management, 2015)^^[[5]](#footnote-5)^^ | Missing | This study was an overview, and assessing the framework´s methodology was out of scope | Jefferies  2016 |
| 26 | Academi wales^^[[6]](#footnote-6)^^. | Missing | This study was an overview, and assessing the framework´s methodology was out of scope | Jefferies  2016 |
| 27 | Sydorchuk’ s list of competencies. | Y | Quality assessment: moderate, I cannot tell or ? | Sydorchuk  2016 |
| 28 | Clinical Leadership Competency Framework | Y | This study was an overview, and assessing the framework´s methodology was out of scope | Stringfellow  2015  &  Quince  2014 |
| 29 | Warde and colleagues’ list of competencies | N | Quality assessment: moderate, I cannot tell or ? | Warde  2014 |
| 30 | Mullan and colleagues´ framework: Domestic and global health equities. | N | Quality assessment: Strong or yes | Mullan  2014 |
| 31 | The Public Health Leadership Competency Framework^^[[7]](#footnote-7)^^ | Y | Quality assessment: Strong or yes | Czabanowska  2014 |
| 32 | Coleman and colleges´ list of program objectives | N | Quality assessment: moderate, I cannot tell or ? | Coleman  2012 |
| 33 | Varkey Coleman and colleges´ list of competencies | Y | Quality assessment: Strong or yes | Varkey  2009 |

Supplementary file 6: Leadership frameworks their thematic scope and target audience (global literature:2009-2022)

| **#** | **Thematic scope: context.** | **Framework^^[[8]](#footnote-8)^^** | **target audience** | **Reference** |
| --- | --- | --- | --- | --- |
| 1 | Refugee and Migrant Health.  Forced migration.  Global migration connect human rights, social development, evidence-based medicine and universal healthcare access. | The Refugee Health Curriculum Framework | UME | Gruner  2022 |
| 2 | NA | AMC Professionalism and Leadership Graduate Domain and Statement (Australia) 2012 | UME | Ross  2021 |
| 3 | NA | Good Medical Practice: A Code of Conduct for Doctors in Australia 2014 | UME | Ross  2021 |
| 4 | NA | Health Leads Australia Framework 2013 | UME | Ross  2021 |
| 5 | NA | Medical Leadership Competency Framework, 2010 | UME | Ross  2021  &  Jefferies  2016 |
| 6 | NA | CanMEDS: Better Standards, Better Physicians, Better Care (Canada) 2015 | UME | Ross  2021 |
| 7 | Missing | Australian Commission on Safety and Quality in Health Care | UME | Ross  2021 |
| 8 | Missing | Leading and Managing Health Services: An Australian Perspective 2015 | UME | Ross  2021 |
| 9 | NA | General Medical Council: Leadership and Management for Doctors (UK) 2012 | UME | Ross  2021 |
| 10 | NA | Kings Fund: Leadership and Leadership Development in Health Care (UK) 2015 | UME | Ross  2021 |
| 11 | Pandemics | The Pandemic Leadership Model. | UME | Bernard  2021 |
| 12 | Quality healthcare. | Rajeh and colleagues´ competencies list | UME | Rajeh  2020 |
| 13 | Homeless and vulnerably housed populations | The Homeless Health Education Framework | UME | Hashmi  2020 |
| 14 | To improve health, health systems and health care. | The Health Systems Science Framework. | 1. UME 2. Pharmacy 3. Nursing 4. Dental 5. Social Work | Wagenschutz 2019 |
| 15 | The health care system | Framework is not provided.  A list of educational objectives is provided | UME ( some of them were doing master or PhD). | Portney 2019 |
| 16 | Healthcare- capstone military medical field practicum.  Military medical leadership | The leader/follower conceptual framework^^[[9]](#footnote-9)^^ | 1. UME 2. Nursing | Barry  2019 |
| 17 | Medical practice- health care | Framework is not provided.  A list of educational objectives is provided | UME | Richard  2019 |
| 18 | Character education to medical students | The 8 core categorical elements of character education. | 1. UME 2. Postgraduate Medical Education ( PGME) | Hur  2019 |
| 19 | Population-based care in a value-based care-delivery system.  To C-suite administrative roles in hospitals and other health care systems | Framework is not provided.  A list of Educational Objectives is provided | 1. UME 2. Residents ( PGME) | Dickerman  2018 |
| 20 | Healthcare systems | Framework is not provided.  A list of competencies is provided | UME | Schmidt-Huber  2017 |
| 21 | Health systems science  To improve quality, outcomes, and costs of health care delivery for patients and populations within medical care systems | Core, cross-cutting, and linking domains for a health systems science (HSS) curricular framework | 1. Undergraduate Medical Education 2. Other health professions schools | Gonzalo 2017 |
| 22 | Leaders, innovators, and scholars in education. | The communities of practice (CoP) framework | 1. UME 2. Residents ( PGME) 3. Fellows ( PGME) | Chen  2017 |
| 23 | NA | Healthcare Leadership Model (NHS Leadership Academy, 2013)- | UME | Jefferies  2016 |
| 24 | NA | Tomorrow’s Doctors (General Medical Council, 2009) | UME | Jefferies  2016 |
| 25 | Missing | the Faculty of Medical Leadership and Management’s (FMLM) Standards for Medical Leadership (Faculty of Medical Leadership and Management, 2015) | UME | Jefferies  2016 |
| 26 | Missing | Academi wales. | UME | Jefferies  2016 |
| 27 | Disciplines «Infectious diseases» and «Epidemiology of Infectious diseases», «Tropical Medicine and Clinical Parasitology» | Framework is not provided.  A list of competencies is provided | UME | Sydorchuk 2016 |
| 28 | NA | Clinical Leadership Competency Framework | UME | Stringfellow,2015.  &  Quince, 2014 |
| 29 | To care for underserved populations | A Framework is not provided.  A list of competencies is provided. | UME | Warde  2014 |
| 30 | Domestic and global health equities. | A name for the framework is not provided  A list of domains with their competencies is provided. | UME | Mullan  2014 |
| 31 | Public Health | The Public Health Leadership Competency Framework | 1. UME 2. Dentistry 3. health sciences 4. microbiology 5. social sciences 6. psychology | Czabanowska2014 |
| 32 | To introduce to careers in academic medicine | Framework is not provided.  A list of program objectives is provided. | UME- dual degree programs | Coleman 2012 |
| 33 | healthcare to address systems challenges and improve the health of the public | Framework is not provided.  A list of competencies is provided | UME | Varkey  2009 |

1. CanMEDS was used to create this CBE (Methodology to contextualise a leadership framework, model, list of competencies/educational objective in the universities) [↑](#footnote-ref-1)
2. Missing competency- based education to teach leadership in undergraduate medical education (Scoping review´s flow diagram: databases, registries, handsearching and consultation.) [↑](#footnote-ref-2)
3. Missing competency- based education to teach leadership in undergraduate medical education (Scoping review´s flow diagram: databases, registries, handsearching and consultation.) [↑](#footnote-ref-3)
4. CanMEDS was used to create this CBE(Methodology to contextualise a leadership framework, model, list of competencies/educational objective in the universities) [↑](#footnote-ref-4)
5. Missing competency- based education to teach leadership in undergraduate medical education (Scoping review´s flow diagram: databases, registries, handsearching and consultation.) [↑](#footnote-ref-5)
6. Missing competency- based education to teach leadership in undergraduate medical education (Scoping review´s flow diagram: databases, registries, handsearching and consultation.) [↑](#footnote-ref-6)
7. Gold Standard framework to converge and diverge with other competency-based education. [↑](#footnote-ref-7)
8. model, framework, or list: competencies or learning objectives. [↑](#footnote-ref-8)
9. The leader/follower conceptual framework embedded the The FourCe framework. [↑](#footnote-ref-9)
