# Supplementary material for: Divergence and Convergence of the Public Health Leadership Competency Framework Against Others in Undergraduate Medical Education: A Scoping Review
Source: Public Health Rev. 2023 Jun 22;44:1605806. doi: 10.3389/phrs.2023.1605806 (PMC10323138; doi:10.3389/phrs.2023.1605806)
Supplement: Supplementary file 4 [file Table3.DOCX]

**Supplementary material 3. Scoping review: eligibility criteria.**

| **PCC** | **Inclusion criteria** | **Exclusion criteria.** |
| --- | --- | --- |
| Population | Literature about UME | Literature that is focused on:  Postgraduate Medical Education  Health workforce education that excludes UME; and  Public Health Workforce education that excludes UME |
| Concept | Literature that covers how to establish, implement, and/or assess leadership frameworks in UME via Competency-Based Education and  Frameworks focus on leadership as the main topic, frameworks that include leadership as a domain, leadership as a competency, or leadership as a learning objective.  Literature covers Undergraduate Medical Students’ aspirations, perceptions, or motivations to create, implement or assess a leadership as framework, domains, competencies or learning objectives. | Literature that fulfils at least one exclusion criterium:  Literature that does not focus on leadership ( e.g. management );  Frameworks that do not mention leadership as the main topic;  Frameworks that do not cover leadership as a domain; and  Literature does not cover how to create, implement, or assess leadership frameworks in UME. |
| Context: | Universities have used a framework. | the leadership frameworks are not used in a university. |
| Types of evidence sources: | Published and grey literature;  Languages: Spanish, Portuguese, and English;  Time frame1970 to 2021. Competency construct starts in the 1970s and 1980s.  References to support time limits.   1. Carraccio, Carol, Wolfsthal, Susan D, Englander, Robert, Ferentz, Kevin, Martin, Christine. Shifting paradigms: From flexner to competencies. Academic medicine. 2002;77(5):361–7 2. Frank, Jason R, Mungroo, Rani, Ahmad, Yasmine, Wang, Mimi, De Rossi, Stefanie, Horsley, Tanya. Toward a definition of competency-based education in medicine: a systematic review of published definitions. Medical teacher. 2010;32(8):631–7. 3. Vasquez, John A, Marcotte, Kayla, Gruppen, Larry D. The parallel evolution of competency‐based education in medical and higher education. The journal of competency-based education. 2021. <https://doi.org/10.1002/cbe2.1234> | Languages that are not mentioned in the inclusion criteria and  The publication is out of the time frame.  Full text is not available. |
| Quality assessment: | Quantitative^[[1]](#footnote-1)^: rate ¨strong¨ or ¨moderate¨.  Qualitative^[[2]](#footnote-2)^: rate ¨yes¨ or ¨I cannot tell¨.  Grey literature^[[3]](#footnote-3)^: ¨yes¨ or ¨? ¨.  Mix methods ^[[4]](#footnote-4)^:rate ¨yes¨ or ¨I cannot tell¨. | Quantitative: rate sections with ¨weak¨.  Qualitative: rate ¨no¨.  Grey literature: ¨no¨.  Mix methods: rate ¨no¨. |

1. The Effective Public Health Practice Project (EPHPP) toolkits, which included the Quality Assessment Tool for Quantitative Studies. [↑](#footnote-ref-1)
2. The Critical Appraisal Skills Programme (CASP) Qualitative Research Checklist. [↑](#footnote-ref-2)
3. The Authority, Accuracy, Coverage, Objectivity, Date, Significance checklist for grey literature. [↑](#footnote-ref-3)
4. The Mixed Methods Appraisal Tool Version 2018 [↑](#footnote-ref-4)
