# Supplementary material for: Divergence and Convergence of the Public Health Leadership Competency Framework Against Others in Undergraduate Medical Education: A Scoping Review
Source: Public Health Rev. 2023 Jun 22;44:1605806. doi: 10.3389/phrs.2023.1605806 (PMC10323138; doi:10.3389/phrs.2023.1605806)
Supplement: Supplementary file 5 [file Table4.DOCX]

**Supplementary material 4. Handsearch**

The procedure had four steps. First, reviewing each article´s reference which was included in this review to include references about thematic scope, and this step was done from January 6^th^ to April 22^nd,^ 2022. Second, determining who cited the manuscript that was included in this review, thereby, Google scholar was used to track literature upwards, and this was done by writing each piece of literature’s tittle in quotation marks, and clicking on the link “ cited by (number)”. The last search was done on January 6^th^ to March 10^th^ 2022. Third, handsearching each journal issue based on the tittles that had been included, if a journal did not have issues; volume was selected. PubMed was consulted by using advanced search and its boxes ( Journal, Volume and issues).

Fourth, searching reviews about teaching leadership across medical education. References were analysed by tittle, abstract and full reading. Inclusion criteria was the same throughout the review, and the exclusion criteria included i) articles that had already been included in the manuscript and had been identified in the handsearching and ii) articles that did not fulfil the quality criteria, and iii) articles that fulfil exclusion criteria.

**Table 1. Handsearching: first, second and third step.**

| **#** | **Article’s title** | **1st step** | **2nd step.** | **3rd step** |
| --- | --- | --- | --- | --- |
| 1 | Leadership and Academic Medicine: Preparing Medical Students and Residents to Be Effective Leaders for the 21st Century. | 24 | 17 | PubMed advanced search("MedEdPORTAL"[Journal]) AND (14[Volume]) (125 tittles) |
| 2 | A first-year leadership programme for medical students. | 10 | 6 | (("Clin Teach"[Journal]) AND (16[Volume])) AND (6[Issue])16, Issue 6 (25 tittles) |
| 3 | Medical Student Consulting: Providing Students Leadership and Business Opportunities While Positively Impacting the Community. | 19 | 4 | ("MedEdPORTAL"[Journal]) AND (15[Volume]) ( 82 tittles) |
| 4 | Medical Student Leader Performance in an Applied Medical Field Practicum. | 54 | 6 | (("Mil Med"[Journal]) AND (184[Volume])) AND (11-12[Issue]) (65 tittles) |
| 5 | Preparing Medical Students to Be Physician Leaders: A Leadership Training Program for Students Designed and Led by Students. | 19 | 6 | ("MedEdPORTAL"[Journal]) AND (13[Volume]) ( 82 tittles) |
| 6 | A medical student leadership course led to teamwork, advocacy, and mindfulness. | 25 | 38 | (("Fam Med"[Journal]) AND (46[Volume])) AND (6[Issue]) ( 14 tittles). |
| 7 | In search for a public health leadership competency framework to support leadership curriculum-a consensus study. | 35 | 66 | (("Eur J Public Health"[Journal]) AND (24[Volume])) AND (5[Issue]) ( 29 tittles) |
| 8 | Leadership curricula and assessment in Australian and New Zealand medical schools. | 36 | 2 | (("BMC Med Educ "[Journal]) AND (21[Volume])) AND (1[Issue]) (626 tittles) |
| 9 | On the road to becoming a responsible leader: A simulation-based training approach for final year medical students. | 39 | 6 | (("GMS J Med Educ "[Journal]) AND (34[Volume])) AND (3[Issue]) ( 8 tittles). |
| 10 | Leadership curriculum in undergraduate medical education: a study of student and faculty perspectives. | 29 | 126 | (("Med Teach"[Journal]) AND (31[Volume])) AND (3[Issue]) ( 24 tittles) |
| 11 | Promoting medical students' reflection on competencies to advance a global health equities curriculum. | 24 | 12 | (("BMC Med Educ "[Journal]) AND (14[Volume])) (296 tittles ) |
| 12 | Preparing students to be academicians: a national student-led summer program in teaching, leadership, scholarship, and academic medical career-building. | 19 | 20 | (("Acad Med"[Journal]) AND (87[Volume])) AND (12[Issue]) (31 tittles) |
| 13 | Contextual Analysis of Stakeholder Opinion on Management and Leadership Competencies for Undergraduate Medical Education: Informing Course Design. | 48 | 1 | ("J Med Educ Curric Dev"[Journal]) AND (7[Volume]) ( 127 tittles.) |
| 14 | Health Systems Science Curricula in Undergraduate Medical Education: Identifying and Defining a Potential Curricular Framework. | 70 | 98 | (("Acad Med"[Journal]) AND (92[Volume])) AND (1[Issue]) ( 34 tittles) |
| 15 | The Health Professions Education Pathway: Preparing Students, Residents, and Fellows to Become Future Educators. | 44 | 64 | (("Teach Learn Med "[Journal]) AND (29[Volume])) AND (2[Issue]) ( 12 tittles). |
| 16 | A student-led curriculum framework for homeless and vulnerably housed populations. | 8 | 2 | (("BMC Med Educ "[Journal]) AND (20[Volume])) AND (1[Issue]) ( 499 tittles). |
| 17 | Identification and evaluation of the core elements of character education for medical students in Korea. | 18 | 3 | (("J Educ Eval Health Prof "[Journal]) AND (16[Volume])) ( 41 tittles) |
| 18 | Leadership and management in UK medical school curricula. | 26 | 24 | (("J Health Organ Manag "[Journal]) AND (30[Volume])) AND (7[Issue]) ( 7 tittles) |
| 19 | Aspects of development of leader creative thinking of medical student at the undergraduate level of medical education. | 5 | 2 | (("Wiad Lek "[Journal]) AND (69[Volume])) AND (6[Issue]) ( 27 tittles) |
| 20 | Defining the structure of undergraduate medical leadership and management teaching and assessment in the UK. | 27 | 30 | (("Med Teach"[Journal]) AND (37[Volume])) AND (8[Issue]) ( 23 tittles) |
| 21 | Leadership and management in the undergraduate medical curriculum: a qualitative study of students' attitudes and opinions at one UK medical school. | 29 | 60 | (("BMJ Open "[Journal]) AND (4[Volume])) AND (6[Issue]) (107 tittles) |
| TOTAL: step 1,2 and 3= 3485 | | 608 | 593 | 2284 |
| Two articles were selected ( 22^nd^ and 23^rd^) and references that provide leadership CBE (24^th^ to 36^th^) . References 22^nd^ and 23^rd^ hand search was performed, as they contained information about new interventions. For the other references about CBE a hand search was not done, as they were part of the same intervention from a previous reference (1^st^ to 21^st^) | | | | |
| 22 An Undergraduate Medical Education Framework for Refugee and Migrant Health: Curriculum Development and Conceptual Approaches. | | 67 | 0 | There was not done as the document did not have volume or issue. “Preprints are preliminary reports that have not undergone peer review”.  (0 tittles) |
| 23. The Pandemic Leadership Model: A Study of Medical Student Values During COVID-19. | | 30 | 1 | International Journal of Medical Students.Vol. 9 No. 4 (2021). Available from [Vol. 9 No. 4 (2021) \| International Journal of Medical Students (ijms.info)](https://www.ijms.info/IJMS/issue/view/v09i04). ( 15 tittles) |
| TOTAL: step 1,2 and 3= 3598 | | 705 | 594 |  |
| 24. Medical Board of Australia. Good Medical Practice: A Code of Conduct for Doctors in Australia. 2014. Available from: <https://www.medicalboard.gov.au/documents/default.aspx?record=WD10%2f1277&dbid=AP&chksum=eNjZ0Z%2fajN7oxjvHXDRQnQ%3d%3d> Accessed on 24^th^ January 2023. | | | | |
| 25. HealthWorkforce Australia. Health Leads Australia Framework 2013. 2013. Available from: [352 (aims.org.au)](https://www.aims.org.au/documents/item/352) Accessed on 24^th^ January 2023. | | | | |
| 26. Academy of Medical Royal Colleges. Medical Leadership Competency Framework: Enhancing Engagement in Medical Leadership Third Edition, July 2010. 2010. Available from: [58652 MLCFO COVER:49688 MLCFO (leadershipacademy.nhs.uk)](https://www.leadershipacademy.nhs.uk/wp-content/uploads/2012/11/NHSLeadership-Leadership-Framework-Medical-Leadership-Competency-Framework-3rd-ed.pdf) Accessed on 24^th^ January 2023. | | | | |
| 27. General Medical Council. Leadership and management for all doctors.2012. Available from: [Leadership and management for all doctors (gmc-uk.org)](https://www.gmc-uk.org/-/media/documents/Leadership_and_management_for_all_doctors___English_1015.pdf_48903400.pdf) Accessed on 24^th^ January 2023. | | | | |
| 28. West, M., Armit, K., Loewenthal, L., Eckert, R., West, T. and Lee, A. Leadership and Leadership Development in Healthcare: The Evidence Base. 2015. London, Faculty of Medical Leadership and Management Available from: [leadership-leadership-development-health-care-feb-2015.pdf (kingsfund.org.uk)](https://www.kingsfund.org.uk/sites/default/files/field/field_publication_file/leadership-leadership-development-health-care-feb-2015.pdf) Accessed on 24^th^ January 2023. | | | | |
| 29. Barry ES, Grunberg NE. A Conceptual Framework to Guide Leader and Follower Education, Development, and Assessment. Journal of Leadership, Accountability and Ethics.2020; 17(1): 127-134. | | | | |
| 30. Callahan, C. W., & Grunberg, N. E. (2019). Military Medical Leadership. In F. G. O'Connor, E. B. Schoomaker, & D. C. Smith (Eds.), Fundamentals of Military Medicine (pp. 51-66). San Antonio, TX: Borden Institute. | | | | |
| 31. Grunberg NE, Barry ES, Callahan CW, Kleber HG, McManigle, Schoomaker EB. A conceptual framework for leader and leadership education and development. [International Journal of Leadership in Education](https://www.tandfonline.com/tedl20). 2019; 22(5): 644–650 | | | | |
| 32 Barry ES, Grunberg NE, Kleber HG, McManigle JE, Schoomaker EB. A four-year medical school leader and leadership education and development program. Int J Med Educ. 2018;9:99-100. | | | | |
| 33. NHS Leadership Academy. (2013). The Healthcare Leadership Model, version 1.0, Leeds: NHS Leadership Academy. Available from: [NHSLeadership-LeadershipModel-colour.pdf (leadershipacademy.nhs.uk)](https://www.leadershipacademy.nhs.uk/wp-content/uploads/2014/10/NHSLeadership-LeadershipModel-colour.pdf) Accessed on 24^th^ January 2023. | | | | |
| 34. General Medical Council. Tomorrow’s Doctors: Outcomes and standards for undergraduate medical education. Available from: [Tomorrow's Doctors 2009 (ub.edu)](http://www.ub.edu/medicina_unitateducaciomedica/documentos/TomorrowsDoctors_2009.pdf) Accessed on 24^th^ January 2023. | | | | |
| 35. NHS Institute for Innovation and Improvement and Academy of Medical Royal Colleges.Medical Leadership Competency Framework, 3rd edition, Coventry: NHS Institute for Innovation and Improvement.2013. Available from: [65589 Leadership Framework_Layout 1 (leadershipacademy.nhs.uk)](https://www.leadershipacademy.nhs.uk/wp-content/uploads/2012/11/NHSLeadership-Framework-LeadershipFramework.pdf) Accessed on 24^th^ January 2023. | | | | |

Consultation process to the corresponding author of the 23 first articles. Authors did not perform a handsearching.

| **#** | **Article’s title** | **Main reference ( the handsearching was done in this article)** |
| --- | --- | --- |
| 36. | Australian Medical Council Limited. [Standards for Assessment and Accreditation of Primary Medical Programs by the Australian Medical Council 2012](https://www.amc.org.au/wp-content/uploads/2019/10/Standards-for-Assessment-and-Accreditation-of-Primary-Medical-Programs-by-the-Australian-Medical-Council-2012.pdf). Available from: [Standards-for-Assessment-and-Accreditation-of-Primary-Medical-Programs-by-the-Australian-Medical-Council-2012.pdf (amc.org.au)](https://www.amc.org.au/wp-content/uploads/2019/10/Standards-for-Assessment-and-Accreditation-of-Primary-Medical-Programs-by-the-Australian-Medical-Council-2012.pdf) Accessed on 24^th^ January 2023. | Ross SJ, Sen Gupta T, Johnson P. Leadership curricula and assessment in Australian and New Zealand medical schools. BMC Med Educ. 2021;21(1):28. |
| 37. | Royal College of Physicians and Surgeon of Canada. Leader. Available from [CanMEDS Role: Leader :: The Royal College of Physicians and Surgeons of Canada](https://www.royalcollege.ca/rcsite/canmeds/framework/canmeds-role-leader-e) Accessed on 24^th^ January 2023. | Gruner D, Feinberg Y, Venables MJ, Hashmi SS, Saad A, Archibald D, Pottie K. An Undergraduate Medical Education Framework for Refugee and Migrant Health: Curriculum Development and Conceptual Approaches. Research Square.2021. Available from: [6e28e5e5-6f8f-4591-8974-b9a9d0b2b759.pdf (researchsquare.com)](https://assets.researchsquare.com/files/rs-781981/v1/6e28e5e5-6f8f-4591-8974-b9a9d0b2b759.pdf?c=1631888403) Accessed on 24^th^ January 2023. |
| 38 | Word document: Medical School: Improving healthcare systems 1, 2, and 3 (MEDADM 5160) | Wagenschutz H, McKean EL, Mangrulkar R, Zurales K, Santen S. A first-year leadership programme for medical students. Clin Teach. 2019;16(6):623-9. |
| 39 | Word document: Medical School University of Michigan 2021 to 22 competencies: word document | Wagenschutz H, McKean EL, Mangrulkar R, Zurales K, Santen S. A first-year leadership programme for medical students. Clin Teach. 2019;16(6):623-9. |
| 40 | Word document: Bushmaster Core Learning Objectives. | Barry ES, Dong T, Durning SJ, Schreiber-Gregory D, Torre D, Grunberg NE. Medical Student Leader Performance in an Applied Medical Field Practicum. Mil Med. 2019;184(11-12):653-60 |
| 41 | Word document: LEAD Pre-Clerkship/B3 Curriculum | Barry ES, Dong T, Durning SJ, Schreiber-Gregory D, Torre D, Grunberg NE. Medical Student Leader Performance in an Applied Medical Field Practicum. Mil Med. 2019;184(11-12):653-60 |
| 42 | Word document: Busmaster Faculty.info. 2021 | Barry ES, Dong T, Durning SJ, Schreiber-Gregory D, Torre D, Grunberg NE. Medical Student Leader Performance in an Applied Medical Field Practicum. Mil Med. 2019;184(11-12):653-60 |
| 43 | Srinivasan M, Li ST, Meyers FJ, Pratt DD, Collins JB, Braddock C, et al. "Teaching as a Competency": competencies for medical educators. Acad Med. 2011;86(10):1211-20. | Chen HC, Wamsley MA, Azzam A, Julian K, Irby DM, O'Sullivan PS. The Health Professions Education Pathway: Preparing Students, Residents, and Fellows to Become Future Educators. Teach Learn Med. 2017;29(2):216-27 |
| 44 | Education, Audiovisual & Culture Executive Agency. Leadership for European Public Health. 2008. | Czabanowska K, Smith T, Könings KD, Sumskas L, Otok R, Bjegovic-Mikanovic V, et al. In search for a public health leadership competency framework to support leadership curriculum-a consensus study. Eur J Public Health. 2014;24(5):850-6. |
| 45 | K.de Jong N, Könings KD, Czabanowska K. The Development of Innovative Online Problem-Based Learning: A Leadership Course for Leaders in European Public Health. Journal of University Teaching & Learning Practice 2014;11 (3).Available [The Development of Innovative Online Problem-Based Learning: A Leadership Course for Leaders in European Public Health](https://files.eric.ed.gov/fulltext/EJ1048875.pdf) Accessed on 24^th^ January 2023. | Czabanowska K, Smith T, Könings KD, Sumskas L, Otok R, Bjegovic-Mikanovic V, et al. In search for a public health leadership competency framework to support leadership curriculum-a consensus study. Eur J Public Health. 2014;24(5):850-6. |
| 46 | L. Könings KD, de Jong N, Lohrmann C, Sumskas L, Smith T, O'Connor SJ, et al. Is blended learning and problem-based learning course design suited to develop future public health leaders? An explorative European study. Public Health Rev. 2018;39:13. | Czabanowska K, Smith T, Könings KD, Sumskas L, Otok R, Bjegovic-Mikanovic V, et al. In search for a public health leadership competency framework to support leadership curriculum-a consensus study. Eur J Public Health. 2014;24(5):850-6. |
| 47 | Katarzyna Czabanowska, Kenneth A. Rethmeier, George Lueddeke, Tony Smith,  André Malho, Robert Otok, Mindaugas, Stankunas. Public Health in the 21^st^ Century:  “Working Differently Means Leading and Learning Differently” (A qualitative study based on interviews with European public health leaders). European Journal of Public Health 2014; doi: 10.1093/eurpub/cku043. | Czabanowska K, Smith T, Könings KD, Sumskas L, Otok R, Bjegovic-Mikanovic V, et al. In search for a public health leadership competency framework to support leadership curriculum-a consensus study. Eur J Public Health. 2014;24(5):850-6. |
| 48 | Katarzyna Czabanowska, André Malho, Peter Schröder-Bäck, Daniela Popa and Genc Burazeri. Do we develop public health leaders? Association between public health competencies and emotional intelligence: a cross-sectional study . BMC Medical Education.2014, 14:83 DOI: 10.1186/1472-6920-14-83. | Czabanowska K, Smith T, Könings KD, Sumskas L, Otok R, Bjegovic-Mikanovic V, et al. In search for a public health leadership competency framework to support leadership curriculum-a consensus study. Eur J Public Health. 2014;24(5):850-6. |

**Table 2. Handsearching: Reviews about Leadership Education in Medical Education.**

| **Number** | **Reviews´ tittle** | **References** |
| --- | --- | --- |
| 1 | Leadership development programs for physicians: a systematic review. | 76 |
| 2 | Training of Leadership Skills in Medical Education | 26 |
| 3 | Faculty development initiatives designed to promote leadership in medical education. A BEME systematic review: BEME Guide No. 19. | 94 |
| 4 | Leadership Training and Undergraduate Medical Education: a Scoping Review | 60 |
| 5 | A systematic review of leadership training for medical students | 43 |
| 6 | A First Step Toward Understanding Best Practices in Leadership Training in Undergraduate Medical Education | 34 |
| Total = 333 | | |

| Total Handsearching: 3598+ 333=3931 |
| --- |
